# The association of ADHD symptoms to self-harm behaviours: a systematic PRISMA review

**DOI:** 10.1186/1471-244X-14-133

**Published:** 2014-05-07

**Authors:** Clare S Allely

**Affiliations:** 1 Institute of Health and Wellbeing, University of Glasgow, RHSC Yorkhill, Glasgow 8SJ G3, Scotland

**Keywords:** ADHD: Attention-Deficit/Hyperactivity Disorder, Self-harm, Self-injurious behaviour, Deliberate self-harm, Suicide-related events

## Abstract

**Background:**

Self-harm is a major public health issue in young people worldwide and there are many challenges to its management and prevention. Numerous studies have indicated that ADHD is associated with completed suicides and other suicidal behaviours (i.e., suicidal attempt and ideation). However, significantly less is known about the association between ADHD and self-harm.

**Method:**

This is the first review of the association between ADHD and self-harm. A systematic PRISMA review was conducted. Two internet-based bibliographic databases (Medline and CINAHL) were searched to access studies which examined to any degree the association between, specifically, ADHD and self-harm.

**Results:**

Only 15 studies were identified which investigated the association between ADHD and self-harm and found evidence to support that ADHD is a potential risk factor for self-harm.

**Conclusion:**

This association raises the need for more awareness of self-harm in individuals with symptoms of ADHD.

## Background

### ADHD and self-harm

Attention-deficit/hyperactivity disorder (ADHD), as defined by DSM-IV [1], is a syndrome that is first manifested in childhood by variable combinations of inattention, hyperactivity and impulsivity. The prevalence of ADHD in childhood is estimated to be approximately 5-7% [2] and ADHD symptoms tend to persist into adulthood [3,4]. ADHD is a mediator of poor outcome such as low self-esteem and poor academic and vocation outcomes [5,6] and one of the major public health problems in modern societies [7].

Nomenclature used to describe acts of self-harm without fatal consequences varies considerably. For this review the terms deliberate self-harm (DSH), self-harm, self-mutilation, self-injury and self-injurious behaviour (SIB) are used interchangeably depending on the use applied in the paper discussed. DSH and SIB represent a significant health problem in the UK and are amongst the most common reasons for emergency hospital admission. Although international variation exists, findings from numerous community-based studies indicate that approximately 10% of adolescents report having self-harmed [8-12]. Self-harm refers to intentional self-poisoning or self- injury, irrespective of type of motive or the extent of suicidal intent [13,14].

### Association between ADHD and suicide

Previous studies have shown a connection between ADHD and attempted and completed suicide in male adolescents and young adults [15-21], suicidal behaviour [22] and suicide ideation in female adolescents [23]. A longitudinal study found that early childhood ADHD is a risk factor for suicidal behaviour between the ages of 9 and 18 [24]. In a clinic- referred sample of female adolescents, Biederman and colleagues found that girls with comorbid ADHD and major depression had more suicidal ideation compared with those with only major depression [25]. Among delinquent juvenile offenders, suicidality relating to depression, ADHD and social phobia were only found among males [26].

A previous systematic review [27] showed an association between ADHD and suicide. Impey [21] conducted a review of the relationship between ADHD and suicidality and found that ADHD symptoms occur more frequently in suicidal populations and may be a reason for completed suicide. In sum, these reviews of the literature showed that there is a positive relationship between ADHD and risk to self.

### Self-harm predicts suicide

Further research is necessary to develop a mean of preventing adolescents from repeating self-harm, since several studies have shown that around 10–15% of children who self-harm are likely to repeat such episodes within a year [28,29], sometimes also proceeding to suicide. Indeed, numerous studies have indicated that self-harm predicts suicide (i.e., [30]) Suicide risk among self-harm patients is hundreds of times higher than in the general population [27].

### Associations of suicide related events and ADHD medications

Although the focus of this systematic review is on ADHD and the outcomes, it is important to include a brief discussion of the crossover with medication and what is currently known. With the treatment of ADHD it is thus important to establish any associations between pharmacological treatments (such as atomoxetine and methylphenidate) and suicide-related events. Atomoxetine (Strattera®) is a selective norepinephrine (noradrenaline) reuptake inhibitor. It is not a stimulant, and is indicated for use in patients with ADHD [31]. Methylphenidate is a central nervous system (CNS) stimulant used to treat ADHD. From the European Union (EU) Supplementary protection certificates (SPCs) for methylphenidate, it is important to highlight that suicidal tendencies have been found to be a contraindication for this medication [32,33]. The US Food and Drug Administration and Health Canada also warned of increased rates of suicidal ideation among children taking atomoxetine in placebo-controlled trials [34]. Based on fourteen identified trials in paediatric patients, Bangs and colleagues (2008) [35] found that, despite being uncommon, suicidal ideation was significantly more frequent in paediatric ADHD patients treated with atomoxetine compared to those treated with placebo [35]. One study found no evidence which suggested an increase in the risk of sudden death associated with stimulants or atomoxetine. However, there was an increased risk of suicide with the treatment [36].

However, a recent meta-analysis, the first focusing on five studies comparing suicide-related events in comparative randomised double-blind atomoxetine and methylphenidate clinical trials, found no significant evidence of a difference in risk between the two treatments [37]. Given the limitations of meta-analyses, acknowledged by the authors [37], further research is required to establish whether there are associations between suicide-related behaviours and specific ADHD treatment medications.

### Present review

There is a need for greater understanding of the factors which contribute to self-harm. Identification of effective prevention initiatives and subsequent treatment strategies aimed at young people and those at particular high risk is clinically imperative. There is an urgent need to identify those populations at risk and to intervene proactively [38]. The purpose of this systematic PRISMA review is to investigate the relationship between ADHD and self-harm, in order to see if self-harm can be considered to be a risk factor. As discussed above this is particularly important given the increased rates of self-harm and the greater risk of these individuals to go on to attempt and/or complete suicide. This is the first review to explore the peer-reviewed literature which has investigated this relationship.

## Method

**Figure 1:**
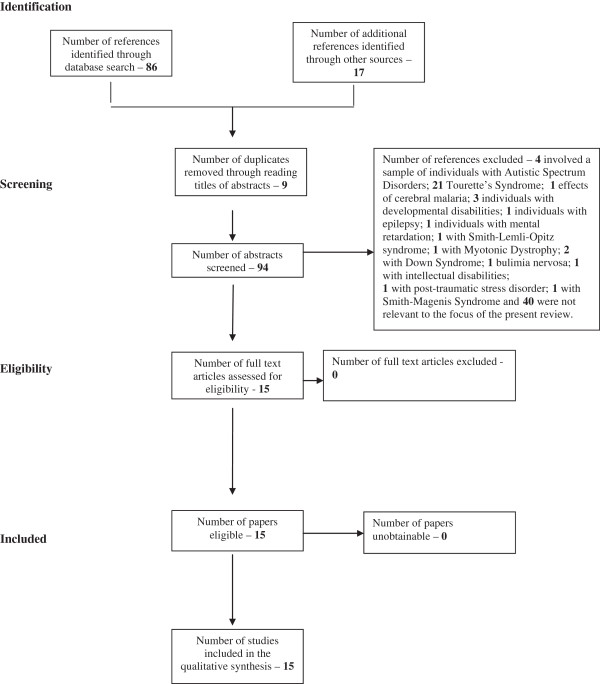
Flow of information through systematic review.

Internet-based bibliographic databases (Medline and CINAHL) were searched to access studies which examined to any degree the association between, specifically, ADHD and self-harm rather than the association between ADHD and completed suicide, suicide attempts and suicide ideation. The search criteria identified below were entered in a number of other databases including PsycINFO. However, the Medline was chosen as the primary database because it returned more relevant articles. CINAHL was also used because it is the definitive research tool for nursing and allied health professionals. The process of eliminating non-relevant papers can be seen in the flowchart (following PRISMA guidelines [39], see Figure 1) below. Duplicates were excluded prior to the retrieval of references. Searches on the two databases were originally conducted on 9th May 2013. The following search criteria were entered into the two databases: [self-harm OR “self harm” OR self-injury OR “self injury” OR self-poisoning OR “self poisoning” OR self-injurious OR “self injurious” OR self-mutilation OR “self mutilation”] AND [ADHD or “Attention-deficit/hyperactivity disorder” or “attention-deficit hyperactivity disorder”]. Medline returned a total of 59 abstracts and CINAHL returned a total of 27. Combining the two search findings (total 86), nine were removed because they were duplicates. In addition to these database searches, numerous permutations of ADHD and self-harm were entered into Google Scholar and thoroughly searched for any additional articles not found in the database searches, for instance, [ADHD AND self harm]; [attention-deficit/hyperactivity disorder]; [ADHD AND self injury]. These searches only returned four additional relevant articles. Numerous references contained in the papers found to be relevant from the database searches were also explored for inclusion in this review.

Abstracts for each reference were obtained and screened using the following criteria:

Inclusion criteria:

1. Human study population

2. Investigated the association between ADHD and self-harm.

Exclusion criteria:

1. Paper not published in English

2. Dissertations

3. Book reviews

4. Studies which investigated a sample that comprised of individuals with a disorder other than ADHD (for instance, individuals with: Autistic Spectrum Disorders; Tourette’s Syndrome; cerebral malaria; developmental disabilities; epilepsy; mental retardation; Smith-Lemli-Opitz syndrome; Myotonic Dystrophy; Down Syndrome; bulimia nervosa; intellectual disabilities; post-traumatic stress disorder and Smith-Magenis Syndrome).

Screening:

In the first stage, papers were rejected which:

•investigated completed suicide, suicide attempts or suicide ideation as this review is specifically interested in the association between ADHD and *self-harm* as even less is known about this association.

•were not published in the English language

For the next stage papers were rejected which:

•were not studies that involved a sample of individuals with ADHD (for instance, numerous studies involved samples of individuals with Tourette’s syndrome).

In addition, review papers and book chapters which were clearly reviews were excluded and if relevant are referred to in the introduction. Full documents were obtained for the remaining records.

## Results

Fifteen studies were found that explored or included an exploration of the association between ADHD and self-harm (one of which was a case study). Table 1 lists the studies that were found to be relevant and details some of the main points for each. In this section, the fifteen studies are split into two sections. The first section includes studies where the sample involved a study population with ADHD and measures of self-harm behaviour were then conducted. The second section includes the studies which examined a population who were hospitalised due to injury and measures of ADHD were then conducted. This was done given the possibility that different results may be obtained depending on this. Under each of these headings, studies are divided depending on whether the samples involved children and/or adolescents under the age of 18 years and adults (those above 18 years of age).

**Table 1:** Lists the studies that were found which explored to any degree the association between ADHD and self-harm and details some of the main points from each (nature of sample; the aim of the study and the main findings)

**Author**	**Samples**	**Aim of the study**	**Findings**
**Ben-Yehuda *et al. *** **2012**[40]	232 ED referrals; 37 (15.9%) children and 195 (84.1%) adolescents.	To investigate the hypothesis that suicidal behaviour in children stems from a different diagnosis other than suicidal behaviour in adolescents.	Findings revealed a diagnostic difference between suicidal children and suicidal adolescents. An act of DSH or ideation was the presenting symptom of 232 ED referrals; this figure comprised 37 (15.9%) children and 195 (84.1%) adolescents. For children, the prevalent diagnoses were ADHD (43.2%), conduct disorders (21.6%), and adjustment disorders (16.2%). For adolescents, the prevalent diagnoses were adjustment disorders (28.7%) and conduct disorders (17.9%) (p < 0.001).
	Children group – under 12 years old.		
	Adolescent group between 12-18 years.		
**Deane and Young (2012)**[41]	8 female participants - 4 from the comorbid group (ADHD/CP; Astrid, Anna, Abigail, and Alison) and four from the control group. (All between 14 & 16 years of age at the time of the interviews).	To investigate the experience of girls growing up with cognitive and social disorders.	2 cases where there was a presentation of both ADHD and self-harm or attempted suicide - Anna and Abigail had attempted suicide and Alison had engaged in extensive self-harm. The authors found that Alison was able to make a clear link between her feelings of emotional isolation, behavioural problems and self-harming behaviour.
		The association between ADHD and self-harm was not the focus of this paper.	
**DiScala *et al. *** **(1998)**[42]	2 groups	To investigate the differences between hospital admitted injuries to children with pre-injury ADHD and injuries to those with no pre-injury conditions (NO).	Compared with the NO children, the children with ADHD were more likely to inflict injury to themselves (1.3% versus 0.1%).
	1) all cases of paediatric trauma that had a pre-injury diagnosis of attention deficit or hyperactive disorder or both ADHD		
			They were more likely to sustain injuries to multiple body regions (57.1% versus 43%), to sustain head injuries (53% versus 41%), and to be severely injured as measured by the Injury Severity Score (12.5% versus 5.4%) and the Glasgow Coma Scale (7.5% versus 3.4%).
	2) all cases of paediatric trauma with no pre-injury condition (NO).		
	ADHD patients (n = 240) to NO patients (n = 21 902), 5 through 14 years of age.		
**Dowson *et al. *** **(2007)**[43]	59 adult patients (mean age: 30.6 years, range 9.8 years) with a DSM-IV diagnosis of ADHD.	To investigate the associations between questionnaire assessments of behavioural features of adults with ADHD and an aspect of neurocognitive performance which has been reported to be impaired in adults with ADHD.	Patients who reported a past history of ‘self-harm' (N = 33) had a significantly worse mean performance on both measures of SWM (p = 0.004, 0.003).
**Dowson *et al. *** **(2010)**[44]	73 male adults with DSM-IV ADHD (aged 18-65 years) and their informants. Impulsive externally directed aggression was endorsed in 29 of the 73 subjects and impulsive autoaggression in 34 subjects.	To investigate the associations between impulsive aggression and ADHD.	Adult ADHD-related impulsivity and hyperactivity predicted temper outbursts ⁄hitting people ⁄throwing, while self-reported adult ADHD-related inattention predicted threats ⁄actual self-harm.
			Impulsive externally directed aggression was endorsed in 29 of the 73 participants and impulsive autoaggression in 34 participants.
**Fulwiler *et al. *** **(1997)**[45]	Inmates were classified as self-mutilators if they had inflicted objectively verifiable bodily injury without either the intent or wish to die (n = 16). Suicide attempters were de- fined as patients whose intention was to die (n = 15). Self-mutilators -mean age 30 years (SD = 7.2). Suicide attempters – mean age 34 years (SD =7.3).	To test the hypothesis that prisoners who injured themselves without intending to die would differ clinically from prisoners who had attempted suicide.	A logistic regression analysis incorporating childhood hyperactivity and affective disorder as covariates found that self-mutilators were 28 times more likely to report childhood hyperactivity.
			The early onset of psychiatric symptoms in self-mutilators was also reflected in the fact that 75 percent (12/16) reported being diagnosed hyperactive as children, compared with only one of the attempters.
**Goodman *et al. *** **(2008)**[46]	AS group - age 8.38 (1.97) years (n = 24).	This study investigated mother and child’s aggression as well as child correlates of suicidal behaviour in two groups—assaultive/suicidal (AS) and assaultive-only (AO) —prepubertal psychiatric inpatients.	AS children were significantly more aggressive and suicidal, five times more likely to engage in serious assaultive behaviour, and almost six times more likely to be diagnosed with ADHD than their AO counterparts. Suicidal behaviour treated as a 5- point dimensional scale in the total sample was associated with child’s aggression, the presence of ADHD, maternal depression, and maternal state anger, but not with child’s depression. Child’s aggression mediated the relation between the presence of ADHD and suicidal behaviour in the total sample.
	AO group - 8.74 (1.82) years (n = 19).		
**Hinshaw *et al. *** **(2012)**[47]	Childhood-ascertained (6-12 years) girls with ADHD (ADHD; n = 140: combined type [ADHD-C] n = 93; inattentive type [ADHD-I] n = 47) plus a matched comparison group (n = 88). 10 year outcomes (age range 17-24 years; retention rate = 95%).	To investigate the 10-year outcomes in girls diagnosed with ADHD in childhood – outcomes investigated were symptoms (ADHD, externalising, internalising), substance use, eating pathology, self-perceptions, functional impairment (global, academic, service utilisation), self-harm (suicide attempts, self-injury), and driving behaviour.	Self-injury was found to be significantly more likely (OR = 4.4) in the ADHD-C group (51%) than the comparison group (19%).
			Self-injury was also found to be more likely in the ADHD-C group compared to the ADHD-I group (29%; OR = 2.5).
			These findings show that self-harmful behaviour predominated in the participants originally diagnosed with ADHD-C.
**Hurtig *et al. *** **(2012)**[48]	Sample derived from a population-based Northern Finland Birth Cohort 1986 (n = 9432). Based on the Schedule for Affective Disorders and Schizophrenia for School-Age Children, Present and Lifetime Version (Kiddie-SADS-PL) interview performed in a subpopulation (n = 457).	To investigate the effect of ADHD on suicidal or self-harm behaviour in adolescents from a general population sample.	Compared with adolescents without ADHD, those with ADHD had more suicidal ideation (57% versus 28%, p < 0.001) and DSH (69% versus 32%, p < 0.001).
	Compared adolescents without ADHD (n = 169) and those with ADHD (n = 104).		
**Izutsu *et al. *** **(2006)**[49]	239 boys (mean age = 14.16 years, SD = 0.67) & 238 girls (14.22, 0.68) from a junior high-school in Kanagawa, Japan	To investigate the status of DSH among junior high-school students, and investigate the relationship between DSH and substance use and childhood hyperactivity.	Overall, 8.00% and 27.70% of males and 9.30% and 12.20% of females reported self-cutting and self-hitting, respectively.
			With respect to the association between DSH and childhood hyperactivity, comparisons of WURS scores between those with and without experience of problematic behaviours revealed that with all problematic behaviours in both genders, scores of those with experience were significantly higher than those without (p < 0.01 except for self-cutting in females, p < 0.05).
**Lam (2002)**[17]	158 with ADD and 46,962 non-ADD individuals between the ages of 16 and 19 years admitted to hospitals due to accidental or self-inflicted injuries in New South Wales, Australia during 1996 to 2000.	To investigate the following: What patient characteristics are associated with the diagnosis of ADD upon admission to the hospital? What types of injury are associated with the diagnosis of ADD among hospitalised young patients? What is the relationship between the diagnosis of ADD and the outcome of hospitalisation due to injury?	Significant association between different causes of injuries, in particular self-inflicted injuries and diagnosis of ADD were found.
**Lam (2005)**[50]	Children and adolescents between the ages of 5 and 15 years admitted to hospital owing to injuries in 2000. 111 individuals with ADD and 18,618 with no ADD.	To investigate the associations between intra-and interpersonal violence and related injuries and the diagnosis of ADD among children and young adolescents.	There were significant associations between suicide and self-harm, injuries owing to assault, and diagnosis of ADD. The odds for self-inflicted injuries were about 8.5 for children diagnosed with ADHD as compared with those without.
**Lynch *et al. *** **(2006)**[51]	12-15 year olds (selected from 8 secondary schools).	To investigate the prevalence rates of psychiatric disorders, suicidal ideation and intent, and parasuicide in a population of Irish adolescents in a defined geographical area.	Investigation of the association between ADHD and self-harm behaviours were not investigated in this paper.
		Investigation of the association between ADHD and self-harm behaviours were not investigated in this paper.	In the ‘at-risk’ group, six (5.9%) had a diagnosis of ADHD and only three (3.2%) had a diagnosis of ADHD in the ‘not at-risk’ group. The estimated prevalence in the entire population (based on a weighted analysis) was 3.7% (95% CI = 0.7-6.7).
**Semiz *et al. *** **(2008)**[52]	105 adult male offenders with Structured Clinical Interview for Axis II Disorders (SCID-II)-based DSM-III-R APD. (Age 20–36 years, mean ± SD = 22.7 ± 2.9 years).	Two-fold:	92 per cent of the participants (n = 97) reported SIB. These included: self-cutting (82%), hitting (51%), burning (37%), and biting (14%). Sixty-five per cent (n = 68) of the subjects had received medical treatment for SIB, indicating the serious and persistent nature of these self-inflicted wounds.
		(1) to define the relationship between DSM-III-R APD and PCL-R-based psychopathy scores with comorbid diagnosis of ADHD (ADHDc) and dimensional ADHD symptoms (ADHDd) in a group of male offenders.	
		(2) To examine the relationship of ADHD measures within the study population with SUD, SIB, and record of suicide attempts and criminal behaviours.	Number of ADHDd symptom criteria endorsed was significantly correlated with frequency of SIB (r = 0.32, p = 0.002). WURS total score was significantly correlated with frequency of SIB (r = 0.38, p < 0.001), number of suicide attempts (r = 0.28, p = 0.011), number of criminal behaviours (r = 0.26, p = 0.016), PCL-R total (r = 0.28, p = 0.016) and Factor 2 scores (r = 0.36, p = 0.002), and negatively correlated with age at onset of SIB (r = – 0.23, p = 0.023). CAARS total score was significantly correlated with frequency of SIB (r = 0.34, p < 0.001) and number of suicide attempts (r = 0.32, p = 0.007).
**Wehmeier *et al. *** **(2008)**[53]	Patients aged 6-17 years with ADHD treated with atomoxetine (target dose 1.2 mg/kg/day). 355 patients completed the 8-week treatment course & 260 patients completed the 24-week treatment course.	To measure changes in items on the PAERS that relate to emotional well-being of children and adolescents with ADHD during treatment with atomoxetine for up to 24 weeks from the perspective of the patient, the parent, and the physician.	421 ADHD patients were treated with atomoxetine - The ten items that reflect emotional well-being were grouped in five dimensions: depressed mood, self-harm, irritability/agitation, drowsiness, and euphoria. The scores of these dimensions decreased over time, both from a patient as well as from a parent and physician perspective.
			Only the dimension self-harm was extremely low at baseline and stayed low over time.

Key

**ADHD-C:** Attention-Deficit/Hyperactivity Disorder - combined type.

**ADHD-I:** Attention-Deficit/Hyperactivity Disorder - inattentive type.

**ADHDd:** ADHD(d) dimensional symptoms by means of Wender Utah Rating Scale (WURS) and Conners Adult ADHD Rating Scale (CAARS) during a 12 month study period (May 2005-May 2006).

**ADD:** Attention Deficit Disorder.

**APD:** Antisocial Personality Disorder.

**AS:** Assaultive/Suicidal.

**AO:** Assaultive-Only.

**CAARS:** Conners Adult ADHD Rating Scale.

**DSH:** Deliberate Self-Harm.

**ED:** Emergency Department.

**NO children:** Injuries to children admitted to hospital with no preinjury conditions (NO).

**PAERS:** Pediatric Adverse Event Rating Scale.

**SIB:** Self-Injurious Behaviour.

**SWM:** Spatial Working Memory.

**WURS:** Wender Utah Rating Scale.

### Studies with a population with ADHD and measures of self-harm behaviour were then conducted

#### Children and adolescent samples

Deane and Young [41] investigated, using Interpretative Phenomenological Analysis of interviews, the experience of eight adolescent girls. Four of whom had a history of ADHD symptoms and conduct disorder problems (ADHD/CP), four did not. However, they report two cases where there was a presentation of both ADHD and self-harm or attempted suicide.

Hinshaw et al. [47] conducted a 10-year prospective follow-up of a female childhood-ascertained (6–12 years) ADHD (n = 140) plus a matched comparison group (n = 88). Ten-year outcomes (age range 17–24 years). Hinshaw et al. [47] assessed variety and frequency of nonsuicidal self-injury (NSSI) using a modification of Claes, Vandereycken, and Vertommen’s [54] Self-Injury Questionnaire (SIQ). Suicide behaviours were investigated using the Barkley Suicide Questionnaire [55]. The family-completed Family Information Profile (FIP) enquired about suicide attempts. For suicide attempts, individuals with ADHD-combined had a higher rate (22%) compared to individuals with ADHD-inattentive (8%) or the comparisons (6%), who did not differ significantly. Interestingly, self-injury was significantly more likely (OR = 4.4) in the ADHD-combined group (51%) compared to the comparison group (19%). Self-harm was also more likely in the ADHD-combined group compared to the ADHD-inattentive group (29%; OR = 2.5).

The idea that ADHD may act as a risk factor for suicidal ideation and DSH was also investigated in a study based on adolescents from a general population sample [48] derived from a population-based Northern Finland Birth Cohort 1986 (n = 9432). Based on the Schedule for Affective Disorders and Schizophrenia for School-Age Children, Present and Lifetime Version (Kiddie-SADS-PL, [56]) interview performed in a subpopulation (n = 457), associations between suicidal behaviour and DSH and the diagnosis of ADHD were studied. Information was also obtained from national registers about deaths related to suicide. Compared with adolescents without ADHD (n = 169), those with ADHD (n = 104) had more suicidal ideation (57% versus 28%, p < 0.001) and DSH (69% versus 32%, p < 0.001). The effect of ADHD on suicidal ideation remained strong (OR = 6.1) after controlling for several other predictors.

The effect of ADHD on substance-use disorder (SUD) as well as other behaviours such as SIB, suicide attempts and criminality is unclear which prompted the next study by Semiz, Basoglu, Oner et al. [52]. A total of 105 adult male offenders with Structured Clinical Interview for Axis II Disorders (SCID-II)-based DSM-III-R antisocial personality disorder (APD) were studied in terms of: (i) psychopathy scores on the Hare Psychopathy Checklist-Revised (PCL-R) [57]; (ii) ADHD(c) diagnostic comorbidity on clinically administered DSM-IV questionnaire; and (iii) ADHD(d) dimensional symptoms by means of Wender Utah Rating Scale (WURS) [58] and Conners Adult ADHD Rating Scale (CAARS [59]), during a 12 month study period (May 2005-May 2006). Most importantly, for the focus of this review, was the finding that the number of ADHD(d) symptom criteria endorsed correlated significantly with frequency of SIB (r = 0.32, p = 0.002). WURS total score was significantly correlated with frequency of SIB (r = 0.38, p < 0.001), number of suicide attempts (r = 0.28, p = 0.011) and negatively correlated with age at onset of SIB (r = − 0.23, p = 0.023). CAARS total score was significantly correlated with frequency of SIB (r = 0.34, p < 0.001) and number of suicide attempts (r = 0.32, p = 0.007).

Wehmeier, Schacht, Lehmann, Dittmann, Silva and March [53] were interested in measuring changes in items on the Pediatric Adverse Event Rating Scale (PAERS) that relate to emotional well-being of children and adolescents with ADHD during treatment with atomoxetine. Patients aged 6–17 years with ADHD were treated with atomoxetine (target dose 1.2 mg/kg/day). The PAERS was used to assess the tolerability of atomoxetine in children and adolescents (n = 421). The ten items that reflect emotional well-being were grouped in five dimensions: depressed mood, self-harm, irritability/agitation, drowsiness, and euphoria. The scores of these dimensions decreased over time. Only the dimension of self-harm was extremely low at baseline and stayed low over time.

#### Adult samples

Dowson, Blackwell, Turner, Harvey, Malhotra, Robbins and Sahakian [43] examined the associations between questionnaire ratings and performance on a computer-administered task of spatial working memory (SWM). Fifty-nine adult patients (mean age: 30.6 years) with a DSM-IV diagnosis of ADHD, and their informants, were asked to complete questionnaires relating to aspects of severity of ADHD. The findings of interest to this particular review were that patients who reported a past history of ‘self-harm’ (n = 33) had a significantly worse mean performance on both measures of SWM (p = 0.004, 0.003).

In a more recent study by the same research group, Dowson and Blackwell [44] investigated impulsive aggression in 73 adults with DSM-IV ADHD adults (mean age of 29). Using questionnaires, they looked at both externally directed aggression and autoaggression. Impulsive autoaggression was identified if one or both of the following Structured Clinical Interview for DSM-IV Personality Disorder (SCID II) questions for BPD criterion 5 were endorsed: “Have you tried to hurt or kill yourself or threatened to do so?”, “Have you ever cut, burned, or scratched yourself on purpose” [60,61]. Impulsive aggression was assessed by ratings of two criteria for borderline personality disorder (BPD), involving hot temper and/or self-harm. Adult ADHD-related impulsivity and hyperactivity was found to predict temper outbursts/hitting people ⁄throwing, while self-reported adult ADHD-related inattention predicted threats/actual self-harm.

### Studies which examined a population who were hospitalised due to injury (or identified by records/self-assessment) and measures of adhd were then conducted

#### Children and adolescents samples

Ben-Yehuda, Aviram, Govezensky, Nitzan, Levkovitz and Bloch [40] found an act of DSH or ideation was the presenting symptom of 232 emergency department referrals out of 905: comprising of 37 (15.9%) children (under 12 years) and 195 (84.1%) adolescents (12–18 years).

DiScala et al. [42] investigated the differences between hospital admitted injuries to children (5–14 years) with preinjury ADHD (n = 240) and injuries to those with no preinjury conditions (NO) (n = 21,902). Findings showed that, compared with the NO children, the children with ADHD were more likely to inflict injury to themselves (1.3% versus 0.1%).

Goodman, Gerstadt, Pfeffer, Stroh and Valdez [46] examined forty-three psychiatrically hospitalised prepubertal children regarding their assaultive and suicidal behaviours and they were subsequently classified into two groups, assaultive/suicidal (AS) and assaultive-only (AO). Mother and child aggression and child correlates of suicidal behaviour in the two groups was investigated. ADHD, child’s aggression, and maternal depression and state anger accounted for 33% of the variance in suicidal-scale scores. Aggression mediated the relation between ADHD and suicidal behaviour. AS children were significantly more aggressive and suicidal, five times more likely to engage in serious assaultive behaviour, and almost six times more likely to be diagnosed with ADHD than their AO counterparts.

Izutsu, Shimotsu, Matsumoto, Okada, Kikuchi, Kojimoto et al. [49] explored the status of DSH among 239 junior high-school boys (mean age = 14.16 years, SD = 0.67) and 238 girls (14.22, 0.68) and investigated the relationship between DSH and substance use and childhood hyperactivity. A self-reporting questionnaire consisting of original questions on self-cutting, self-hitting, and tobacco and alcohol were used in addition to the Wender Utah Rating Scale (WURS) for assessing childhood hyperactivity. With respect to the association between DSH and childhood hyperactivity, comparisons of WURS scores between those with and without experience of problematic behaviours revealed that with all problematic behaviours in both genders, scores of those with experience were significantly higher than those without (p < 0.01 except for self-cutting in females, p < 0.05).

Lam [17] found a four-fold higher likelihood of having a diagnosis of ADD for children and adolescents hospitalised for suicide attempts and self-harm based on a population-based epidemiological design which analysed data routinely collected on patients hospitalised due to injuries.

Lam [50] investigated the associations between intra-and interpersonal violence and related injuries and the diagnosis of attention deficit disorder (ADD) among children and young adolescents. This was a population-based epidemiological study that analysed data routinely collected on hospitalised patients owing to injuries. Children and adolescents (between 5–15 years) were identified from the ISC data bank by the selection criteria of having a diagnosis of external causes of injury and poisoning according to the International Classification of Diseases 9th Revision-Clinical Modification (ICD-9-CM) [62]. Patients with comorbidity of ADD were further identified from this data set by the ICD-9-CM. The likelihood of being diagnosed with ADD was about four times higher (OR = 3.76, 95% CI = 1.73-8.15) for children and adolescents hospitalisations owing to suicide and self-harm. Patients who were admitted to hospitals owing to suicide/self-harm were six times (OR = 6.27, 95% CI = 2.76-14.26) and three times (OR = 3.05, 95% CI = 1.31-7.06) more likely to be diagnosed with ADD, respectively, as compared with other causes of injury. The odds for self-inflicted injuries were about 8.5 for children diagnosed with ADHD compared to individuals without ADHD.

Lynch, Mills, Daly and Fitzpatrick [51] investigated the prevalence rates of psychiatric disorders, suicidal ideation and intent and parasuicide in a population of Irish adolescents aged 12–15 years. 19.4% of the 723 screened were identified as being ‘at risk’ and this ‘at risk’ group were interviewed along with a comparison sample matched for gender, school and school year. In the ‘at-risk’ group, six (5.9%) had a diagnosis of ADHD and only three (3.2%) had a diagnosis of ADHD in the ‘not at-risk’ group. The estimated prevalence in the entire population (based on a weighted analysis) was 3.7% (95% CI = 0.7-6.7).

#### Adults samples

Fulwiler, Forbes, Santangelo and Folstein [45] tested the hypothesis that prisoners who injured themselves without intending to die would differ clinically from prisoners who had attempted suicide. Fifteen patients reported that they had attempted to take their own lives, while 16 reported other reasons for harming themselves. The findings most relevant to the focus of this review was that of self-mutilation with a history of childhood hyperactivity (12/16 versus 1/15 suicide attempters). Five remembered being treated with Ritalin and several reported that illicit stimulant drugs (cocaine. amphetamines) had a calming effect and helped them concentrate. Self-mutilators were 28 times more likely to report childhood hyperactivity, and suicide attempters were 21 times more likely to be diagnosed with major affective disorder.

## Discussion

This review identified 15 studies which investigated the association between ADHD and self-harm (one of which was a case study design). All of these studies indicate an association between ADHD and self-harm which suggests that ADHD may be a potential risk factor for self-harm.

It is important to consider the age of the individuals with ADHD in the studies which have been identified by this review (which investigated the association between ADHD and self-harming behaviours) given the literature which suggests that ADHD symptoms can sometimes change with age. Although ADHD symptoms frequently persist over time [63], maturation has been found to have a significant positive effect on ADHD symptoms in many children [64]. These findings have resulted in the hypothesis that ADHD is associated with a delay as opposed to an abnormal brain development [65,66]. However, few studies have examined the persistence of ADHD from childhood to adulthood [67,68]. This lack of rigorous research is surprising, given the significant impact that ADHD frequently has on the individual. For instance, ADHD diagnosed at school age increases the risk for antisocial development, drug misuse, pathological aggression, and social and academic exclusion by a factor of five to ten compared to the general population [69-71].

It is also important to highlight the gender in the studies identified by this review given the gender differences seen in individuals with ADHD [72]. The existing literature shows that although the gender difference in childhood is quite large, in adult samples this difference diminishes or disappears. Studies investigating gender differences, indicates that girls may consistently be under-identified and under-diagnosed and it is suggested that differences in the expression of ADHD between the genders might be one explanation for this [73-76]. Females with ADHD are reported to have less hyperactive/impulsive symptoms and more inattentive symptoms compared to males with ADHD [73,77,78]. Diagnosis of the inattentive subtype also appears to be more common in females with ADHD [79]. Boys with ADHD appear to exhibit more externalising disorders compared to boys without ADHD. Females, on the other hand, tend to exhibit more internalising disorders compared to girls without ADHD [73,74,78,80] and to their male counterparts [80].

Looking at the gender in the group of interest across all fifteen studies it is clear that there are much higher numbers of males than females. This can be seen more clearly in Table 2. Studies which include greater levels of females are required. The small number of females in a large proportion of studies is limiting the power and the ability to find any significant association between ADHD and self-harming behaviours.

**Table 2:** The number of males and females in each group of interest across all 15 studies identified in this review

**Study**	**Male**	**Female**
Ben-Yehuda et al. [40]	Does not specify for the DSH individuals.	Does not specify for the DSH individuals.
	Of the 39 suicidal children, 25 were males (64%).	Of the 39 suicidal children, 14 were females (36%).
	Of the 227 suicidal adolescents, 58 (26%) were males.	Of the 227 suicidal adolescents, 169 (74%) were females.
Deane and Young (2012) [41]	No males (n = 0)	ADHD (n = 4)
DiScala et al. (1998) [42]	ADHD (n = 211)	ADHD (n = 28)
Dowson et al. (2007) [43]	ADHD (n = 43)	ADHD (n = 16)
Dowson et al. (2010) [44]	ADHD (n = 73)	No females (n = 0)
Fulwiler et al. [45]	Self-mutilators (n = 15)	Self-mutilators (n = 1)
	Suicide-attempters (n = 11)	Suicide-attempters (n = 4)
Goodman et al. [46]	Assaultive/suicidal (83.3% of 24).	Assaultive/suicidal (16.7% of 24)
	Assaultive-only (89.5% of 19)	Assaultive-only (10.5% of 19)
Hinshaw et al. [47]	No males (n = 0)	ADHD (n = 140)
Hurtig et al. (2012) [48]	ADHD and DSH (n = 15)	ADHD and DSH (n = 30)
	Suicidal Acts & ADHD (n = 4)	Suicidal Acts & ADHD (n = 4)
Izutsu et al. (2006) [49]	DSH (n = 239)	DSH (n = 238)
Lam (2002) [17]	ADD and ED admission for injury (n = 125). * States that of the types of injuries they looked at, there were 59 cases of suicide/self-harm but does not specify the gender of this group of injuries.	ADD and ED admission for injury (n = 33). See male column for more detail.
Lam (2005) [50]	ADD (n = 97)	ADD (n = 33)
Lynch et al. (2006) [51]	‘At-risk’ of psychiatric disorder (n = 67)	‘At-risk’ of psychiatric disorder (n = 73)
Semiz et al. (2008) [52]	ADHD (n = 68.25) (65% of 105)	No females (n = 0)
Wehmeier et al. (2008) [53]	ADHD (n = 338) (80.3%)	ADHD (n = 83) c19.7%)

The majority of the studies used samples of children (below the age of 18 years). Only three studies used a sample of adults (older than 18 years) [43-45], of which two were by the same research group [43,44]. Given what has just been described about the potential effect of age on ADHD symptoms, more studies are required which use samples to include older people in order to elucidate the effect of ADHD over the course of their lifetime.

From the 15 identified studies in this review, seven were studies using a population with ADHD and measures of self-harming behaviour were then conducted [41,43,44,47,48,52,53]. The remaining studies investigated a population who were hospitalised due to injury (or identified by records/self-assessment) and measures of ADHD were then conducted [17,40,42,45,46,49-51]. Three of the 15 studies discussed in this systematic review were identified as the most relevant and methodologically reliable [42,47,48]. DiScala et al. [42] investigated the differences between hospital admitted injuries to children with pre-injury ADHD and injuries to those with no pre-injury conditions (NO). Compared with the NO children, the children with ADHD were more likely to inflict injury upon themselves (1.3% versus 0.1%). The importance of early identification of individuals at greater risk of self-harm is further validated by the findings of Hinshaw et al. [47] which showed that girls with childhood ADHD maintain marked impairment by early adulthood (including higher rates of suicide attempts and self-injury). Hinshaw et al. [43] found that girls with childhood-diagnosed ADHD continued to display higher rates of ADHD and comorbid symptoms and exhibited higher rates of suicide attempts and self-injury compared with the comparison sample. Self-harm behaviour predominated in the participants originally diagnosed with ADHD-combined. Hinshaw et al. [47] indicated that individuals with the ADHD combined type are at even greater risk of self-harm behaviours which merits further attention. Lastly, Hurtig et al. [48] investigated the effect of ADHD on suicidal or self-harm behaviour in adolescents from a general population sample and found that, compared with adolescents without ADHD, those with ADHD had more suicidal ideation (57% versus 28%) and DSH (69% versus 32%)”.

The importance of further research into the association between ADHD and self-harm is further highlighted by the studies (outwith this review) which have investigated the increased rates of suicide in individuals with ADHD. For instance, Barbaresi, Colligan, Weaver, Voigt, Killian and Katusic (2013) [81] investigated long-term outcomes of ADHD in a population-based sample of childhood ADHD cases (n = 367) and controls, who were all prospectively assessed as adults. Importantly, findings revealed that childhood ADHD is a chronic health problem, with significant risk for mortality, persistence of ADHD, and long-term morbidity in adulthood. The cause-specific mortality for suicide only was significantly higher among ADHD cases (standardised mortality ratios, SMR, 4.83; 95% CI, 1.14–20.46; P = .032) compared to non-ADHD controls from the same birth cohort [81].

### Limitations

The conclusions that can be made regarding the strength of association between ADHD and self-harm are limited due to the relatively small amount of studies that have been conducted to date. In addition, the majority of these studies use small populations and some do not contain (or report on) control group findings. Publication bias is likely to be present as studies reporting no correlation are unlikely to be published [21]. Differing methodologies and differing measurement tools used across all the studies are a further complication [32]. For instance, in one of the studies found in this review, childhood hyperactivity was inferred only by self-report (as opposed to potentially more robust reports such as clinical assessments and/or parental reports) which the authors acknowledge in their paper [49].

Another limitation is the fact that data for the studies were collected retrospectively and then analysed for suicidal related events which exposes the studies to a degree of confounding bias, particularly in the studies where, for example, the Columbia Classification Algorithm for Suicide Assessment (C-CASA) (the standardised suicidal rating system) and the Computerized Suicide Risk Scale (CSRS) have not been delivered. Mapping any event to a specific code is possible, however, the potential for error is greater than if the scales were used prospectively. The use of these scales are often mandatory in trials. Additionally, the ability to separate self-harm as an entity from a suicide attempt is not always easy which is a potential confounding bias. In particular, Silverman, Berman, Sanddal, O’Carroll and Joiner (2007) [82] emphasised the potential complication with trying to separate self-harm and suicide attempt into two independent categories by highlighting that self-injurious intent and suicidal intent can be present simultaneously in an individual [82]. SIB and suicidal behaviours exists along the same continuum, with SIB representing a lesser form [83,84]. Research has also found that 28% to 41% of individuals who engage in SIB report suicidal thoughts at the time they were engaged in self-injury [85].

Lastly, the influence of comorbidity is difficult to disentangle from the main findings, particularly for substance misuse and delinquency, prompting the need for further research using younger populations and those without co-morbidity [21].

### Future directions and clinical implications

One study has shown some understanding of this relationship in their treatment strategies. Carminati, Deriaz and Bertschy [86] had good experiences with venlafaxine in the treatment of SIB and ADHD-like symptoms in patients with pervasive developmental disorders. This review highlights the need for clinicians to recognise the ADHD population as being at increased risk of self-harm. Children with ADHD pose particular problems of engagement with child mental health services which is another issue that needs to be addressed (a review of this is outside the scope of this review). Also, as the review by James [32] highlights, there is a need for clinicians to also recognise that the ADHD population is at increased risk of suicide attempt and completed suicide. The clinical implications of the main findings from the present review is that clinicians need to routinely screen for suicide attempts and self-harm in ADHD subjects, including the younger population which may introduce new ways of further reducing the youth suicide and self-harm rate [32]. This review also indicates that there needs to be a revision of the standardised ADHD rating scales since they generally do not enquire about self-harm and risk to self and others. This review clearly emphasises the need for inclusion of these aspects during routine assessment (either in questionnaires, clinical assessment or both). Effective screening requires an integration of all the currently known predictors of self-harm (and this review strongly highlights the need for symptoms of ADHD to be one of them) and further research to identify any others. One study discussed in this review also supports the idea that there needs to be distinctive screening procedures for each of these sub-types [46].

## Conclusions

Despite the contribution to our knowledge of self-harm behaviours to date, we remain unclear as to exactly why individuals engage in these behaviours and, even more importantly, we do not have in place effective methods of accurately predicting or reducing these behaviours as a consequence. Therefore, it is vitally important that risk factors for self-harm (such as symptoms of ADHD) are recognised and identified to produce more reliable risk level indicators.

## Competing interests

The author declares that she has no competing interests.

## Pre-publication history

The pre-publication history for this paper can be accessed here:

http://www.biomedcentral.com/1471-244X/14/133/prepub
